# Critical shortage of capacity to deliver safe paediatric surgery in sub-Saharan Africa: evidence from 67 hospitals in Malawi, Zambia, and Tanzania

**DOI:** 10.3389/fped.2023.1189676

**Published:** 2023-05-31

**Authors:** Jakub Gajewski, Chiara Pittalis, Eric Borgstein, Leon Bijlmakers, Gerald Mwapasa, Mweene Cheelo, Adinan Juma, Muskan Sardana, Ruairi Brugha

**Affiliations:** ^1^ Institute of Global Surgery, Royal College of Surgeons in Ireland, Dublin, Ireland; ^2^ Centre for Global Surgery, University of Stellenbosch, Cape Town, South Africa; ^3^ Department of Surgery, College of Medicine Malawi, Blantyre, Malawi; ^4^ Department of Health Evidence, Radboud University Medical Center, Nijmegen, The Netherlands; ^5^ Surgical Society of Zambia, University Teaching Hospital Lusaka, Lusaka, Zambia; ^6^ East Central and Southern Africa Health Community, Arusha, Tanzania

**Keywords:** sub-Saharan Africa, surgery, pediatric surgery, district hospital, rural Africa, surgical capacity

## Abstract

**Introduction:**

Paediatric surgical care is a significant challenge in Sub-Saharan Africa (SSA), where 42% of the population are children. Building paediatric surgical capacity to meet SSA country needs is a priority. This study aimed to assess district hospital paediatric surgical capacity in three countries: Malawi, Tanzania and Zambia (MTZ).

**Methods:**

Data from 67 district-level hospitals in MTZ were collected using a PediPIPES survey tool. Its five components are procedures, personnel, infrastructure, equipment, and supplies. A PediPIPES Index was calculated for each country, and a two-tailed analysis of variance test was used to explore cross-country comparisons.

**Results:**

Similar paediatric surgical capacity index scores and shortages were observed across countries, greater in Malawi and less in Tanzania. Almost all hospitals reported the capacity to perform common minor surgical procedures and less complex resuscitation interventions. Capacity to undertake common abdominal, orthopaedic and urogenital procedures varied—more often reported in Malawi and less often in Tanzania. There were no paediatric or general surgeons or anaesthesiologists at district hospitals. General medical officers with some training to do surgery on children were present (more often in Zambia). Paediatric surgical equipment and supplies were poor in all three countries. Malawi district hospitals had the poorest supply of electricity and water.

**Conclusions:**

With no specialists in district hospitals in MTZ, access to safe paediatric surgery is compromised, aggravated by shortages of infrastructure, equipment and supplies. Significant investments are required to address these shortfalls. SSA countries need to define what procedures are appropriate to national, referral and district hospital levels and ensure that an appropriate paediatric surgical workforce is in place at district hospitals, trained and supervised to undertake these essential surgical procedures so as to meet population needs.

## Introduction

The lack of access to surgical care in low-and middle-income countries (LMIC) requires urgent attention. Currently, around 1.7 billion children and adolescents do not have access to potentially life-saving surgeries (1). In sub-Saharan Africa (SSA), paediatric disease is rife, with under-five mortality rates 15 times higher than in high-income countries (HIC) (2). Where paediatric surgery is available, outcomes in SSA are considerably worse than in HIC for common conditions in infants, such as repair of gastroschisis (75.5% mortality in SSA vs. 2.0% in HIC) and repair of anorectal malformation (11.2% mortality in SSA vs. 2.9% in HIC). Common surgical procedures such as paediatric hernia repair in SSA also result in higher and avoidable mortality (3).

In SSA, shortages have been reported for all members of the specialist surgical team (4). For example, in Tanzania, the current ratio of surgeons, anaesthesiologists and obstetricians (SAO providers) is 0.46 compared to the recommended ratio of 20 SAOs per 100,000 population (5). In SSA, there is an estimated one paediatric surgeon for every six million children aged 0 to 14 years (6). Outcomes for specific paediatric surgical conditions in LMICs correlate with paediatric surgical workforce density, as demonstrated in a recent systematic review, where a paediatric surgical workforce density greater than 0.4 per 100,000 children below 5yrs was significantly correlated with increased odds of survival in surgery undertaken for gastroschisis, oesophageal atresia, intestinal atresia and typhoid perforation (7). In many countries in SSA, front-line paediatric healthcare is provided by medical staff with no specialist paediatric training (8).

Malawi, Tanzania and Zambia (MTZ) are three countries in SSA that have a combined population of 92 million, of which at least 50% are under the age of 18 (9). A recent study in Malawi reported that 26% of children (approximately 2 million) live with a condition treatable with surgery (10). Similar data for Zambia and Tanzania has not yet been collected.

In order to inform the scale-up of paediatric surgical services, available data on existing services and resources at district hospitals in SSA need to be collected and analysed to enable local authorities to make informed decisions on how best to maximise the utilisation of scarce resources to meet population needs (11). However, little systematic evidence has been collected at the facility level (12), and no study has been done comparing the capacity of district facilities across countries. This study aimed to examine and compare the capacity of district hospitals in three SSA countries (MTZ) to provide paediatric surgical care, using tools specifically designed to measure the capacity components (13) so as to establish a baseline and inform policy and planning at country level.

## Methods

We conducted a cross-sectional study using the PediPIPES surgical capacity assessment tool (13) which is an extension of the PIPES tool (14). Several studies have endorsed PIPES as a valid and reliable instrument to measure surgical capacity in resource-constrained settings (15). The PediPIPES tool measures a health facility's capacity to deliver emergency and essential surgical care (EESC) to infants and children under 18 years (13, 16) by examining the availability of procedures, personnel, infrastructure, equipment and supplies. The PediPIPES survey components include the following numbers of variables: procedures 46, personnel 7, infrastructure 17, equipment 22, and supplies 26 variables. Numbers are recorded of available staff, incubators, paediatric ventilators, paediatric beds and operating rooms. For other variables, a binary value of 1 is given where the item is reported adequate or always available, and where a surgical procedure is actually conducted at the hospital and a score of 0 if the item is unavailable/inadequate or where a surgical procedure is not conducted. The tool has no maximum score for the number of available personnel, operating rooms, incubators or ventilators. An overall hospital surgical capacity index is computed based on individual variable scores, which is then used to compare facilities and monitor trends over time.

### Data collection

Data were collected from 67 district level hospitals (DHs) across the three countries between October 2018 and July 2019 as part of the SURG-Africa project (17). The detailed study design and sampling strategy have been reported elsewhere (17). In Malawi, on request of local authorities, faith-based facilities were excluded from the study sample. Data from each hospital were collected at country workshops organised for this purpose. To complete the PediPIPES survey, a minimum of two core surgical team members per facility responded to increase the validity and reliability of the answers and minimise recall bias reported in other studies done using the PIPES tool (15, 18). The survey was administered in English. The local researchers used local vernacular to explain or clarify questions where needed. Questions were read out aloud by one of a team of full-time local and international project researchers. The hospital respondents were asked to discuss each item and provide an agreed response (18). The collected data were transferred from the completed PediPIPES survey instrument into Excel sheets for data processing and analysis.

### Data analysis

Numbers of beds per hospital were excluded from the analysis as most DLHs do not have a formal bed establishment for paediatric surgical cases. Also, respondents had difficulty agreeing on the actual number of paediatric surgical beds and such cases are often found in surgical or medical wards alongside adult post-operative cases. Comparisons between faith-based (church-owned) and government-owned hospitals were done in Zambia and Tanzania. The analysis of self-reported capacity (defined as having been trained) to perform paediatric procedures was done using the list of procedures on the survey tool. These were later grouped into eight categories. Analysis produced a total PediPIPES score for MTZ as per the survey manual (14) and a two-tailed analysis of variance (ANOVA) test was used to explore differences and cross-country comparisons. Descriptive statistics were computed for individual variables. Analysis was performed using SPSS-IBM v25 (IBM Corp, Armonk, NY).

### Ethical considerations

Ethical approval was granted by the Research Ethics Committee (REC) of the Royal College of Surgeons in Ireland, the project consortium lead, under approval No. REC 1417. In the implementation countries, ethical approval was received from the College of Medicine REC in Malawi (approval No. P.05/17/2179), the University of Zambia Biomedical REC (approval No. 005-05-17), the Kilimanjaro Christian Medical College Research Ethics and Review Committee (approval No. CRERC 2026), and the National Institute for Medical Research in Tanzania (approval No. NIMR/HQ/R.8a/Vol. IX/2600).

## Results

The overall PediPIPES Index scores were similar across the three countries. Zambia (Mean [M] = 5.84, range: 4.92–7.29, standard deviation [SD] = 0.67) and Malawi (M = 5.85, range = 3.73–8.14, SD = 1.02) scored the lowest on average. Tanzania scored the highest (M = 6.24, range: 4.07–7.63, SD = 0.95). Differences between countries were not statistically significant (*p* = 0.231). The mean index score for faith-based hospitals in Tanzania and Zambia combined was 6.25 (range = 5.00–7.63, SD = 0.76), compared with 5.91 (range = 3.73–8.14, SD = 0.94) for government owned hospitals. This difference was not statistically significant (*p* = 0.203).

### Procedures

Almost all district hospitals across the three countries reported having the skills to perform common minor emergency and elective procedures, including resuscitation, suturing, wound debridement, burns management, incision and drainage, and male circumcision (Table 1). Skills to perform laparotomies, insertion of gastro-intestinal tube, chest tube insertion, repairs of testicular torsion and paediatric hernia repairs were less commonly reported in Tanzania. Availability of skills needed for closed fractures was commonly reported (87%–96%) across all hospitals, with management of open fractures, osteomyelitis and amputations more commonly done in Malawi. Surveyed clinicians reported no skills for more advanced or complex resuscitation techniques (e.g., tracheostomy or thoracotomy).

**Table 1:** Procedures for which availability of skills to perform were reported at district hospitals.

Procedure category	Procedure	Malawi (*n* = 22 DHs)	Zambia (*n* = 21 DHs)	Tanzania (*n* = 24 DHs)	TOTAL (*n* = 67)
		*N* (%)	*N* (%)	*N* (%)	*N* (%)
Minor procedures	Resuscitation	22 (100%)	21 (100%)	24 (100%)	67 (100%)
	Suturing	22 (100%)	21 (100%)	24 (100%)	67 (100%)
	Wound debridement	22 (100%)	21 (100%)	23 (95.8%)	66 (98.5%)
Surgical techniques	Incision and drainage of abscess	22 (100%)	21 (100%)	24 (100%)	67 (100%)
	Laparotomy	22 (100%)	20 (95.2%)	20 (83.3%)	62 (92.5%)
Trauma	Laparoscopic surgery	0 (0%)	0 (0%)	0 (0%)	0 (0%)
	Burn management	22 (100%)	21 (100%)	24 (100%)	67 (100%)
	Skin grafting	9 (40.9%)	5 (23.8%)	4 (16.7%)	18 (26.9%)
Airway management	Tracheostomy	3 (13.6%)	3 (14.3%)	3 (12.5%)	9 (13.4%)
	Chest tube insertion	22 (100%)	19 (90.5%)	13 (54.2%)	54 (80.6%)
	Removal of airway and oesophageal foreign body	22 (100%)	14 (66.7%)	14 (58.3%)	50 (74.6%)
	Thoracotomy	0 (0%)	0 (0%)	0 (0%)	0 (0%)
	Repair of oesaphageal atresia	0 (0%)	0 (0%)	0 (0%)	0 (0%)
Gastrointestinal	Non-operative reduction of intussusception	4 (18.2%)	5 (23.8%)	4 (16.7%)	13 (19.4%)
	Appendectomy	15 (68.2%)	17 (81%)	24 (100%)	56 (83.6%)
	Resection of solid abdominal mass	15 (68.2%)	8 (38.1%)	9 (37.5%)	32 (47.8%)
	Paediatric abdominal wall defect repair	2 (9.1%)	2 (9.5%)	1 (4.2%)	5 (7.5%)
	Bowel resection and anastomosis	14 (63.6%)	9 (42.9%)	13 (54.2%)	36 (53.7%)
	Repair imperforate anus	4 (18.2%)	3 (14.3%)	2 (8.3%)	9 (13.4%)
	Ladd procedure	4 (18.2%)	4 (19%)	2 (8.3%)	10 (14.9%)
	Rectal biopsy	4 (18.2%)	4 (19%)	7 (29.2%)	15 (22.4%)
	Pull-through for hirschsprung disease	0 (0%)	0 (0%)	0 (0%)	0 (0%)
	Insertion of g-tube	18 (81.8%)	19 (90.5%)	14 (58.3%)	51 (76.1%)
	Repair of intestinal atresia	0 (0%)	0 (0%)	0 (0%)	0 (0%)
	Creation of intestinal stoma	6 (27.3%)	3 (14.3%)	0 (0%)	9 (13.4%))
	Closure of intestinal stoma	6 (27.3%)	3 (14.3%)	0 (0%)	9 (13.4%)
	Pyloromyotomy	0 (0%)	0 (0%)	0 (0%)	0 (0%)
Urogenital	Male circumcision	22 (100%)	21 (100%)	24 (100%)	67 (100%)
	Paediatric hernia repair	20 (90.9%)	18 (85.7%)	14 (58.3%)	52 (77.6%)
	Orchiopexy	8 (36.4%)	9 (42.9%)	8 (33.3%)	25 (37.3%)
	Repair of testicular torsion	16 (72.7%)	14 (66.7%)	10 (41.7%)	40 (59.7%)
	Repair of imperforate hymen	19 (86.4%)	10 (47.6%)	11 (45.8%)	40 (59.7%)
	Ovarian cystectomy	22 (100%)	21 (100%)	24 (100%)	67 (100%)
Orthopaedics	Splinting	20 (90.9%)	19 (90.5%)	22 (91.7%)	61 (91.0%)
	Casting	22 (100%)	21 (100%)	21 (87.5%)	64 (95.5%)
	Traction closed fracture	22 (100%)	19 (90.5%)	17 (70.8%)	58 (86.6%)
	Open Treatment of fracture	19 (86.4%)	5 (23.8%)	10 (41.7%)	34 (50.7%)
	Management of osteomyelitis	21 (95.5%)	15 (71.4%)	19 (79.2%)	55 (82.1%)
	Amputation	22 (100%)	16 (76.2%)	15 (62.5%)	53 (79.1%)
	Club foot repair (non-surgical)	16 (72.7%)	11 (52.4%)	10 (41.7%)	37 (55.2%)
	Repair spina bifida	0 (0%)	0 (0%)	0 (0%)	0 (0%)
	Contracture release	13 (59.1%)	6 (28.6%)	17 (70.8%)	36 (53.7%)
Anaesthetics	Regional anaesthesia blocks	20 (90.9%)	16 (76.2%)	10 (41.7%)	46 (68.7%)
	Spinal anaesthesia	22 (100%)	21 (100%)	23 (95.8%)	66 (98.5%)
	Ketamine anaesthesia	22 (100%)	21 (100%)	24 (100%)	67 (100%)
	General anaesthesia	22 (100%)	18 (85.7%)	21 (87.5%)	61 (91.0%)

Few or no district hospitals in all three countries reported capacity to undertake surgery on major congenital malformations, such as oesophageal atresia, intestinal atresia, Hirschsprung's disease, pyloromyotomy or spina bifida. A small number of hospitals reported having capacity to manage intussusception, undertake repairs of imperforate anus and orchidopexy, and creation and closure of intestinal stomas. While there was variation in the reported procedures, district hospitals in Malawi were more likely to have such skills, with resection of abdominal mass undertaken in almost twice as many district hospitals in Malawi compared to Zambia (68% vs. 38%). Capacity to perform emergency abdominal surgeries such as laparotomy and appendectomy was commonly reported (93% and 84%, respectively), with around half of all hospitals across MTZ reporting capacity to undertake bowel resection and anastomosis. Most district hospitals reported capacity to deliver general, spinal and ketamine anaesthesia, with less than half of district hospitals performing regional blocks in Tanzania—see Table 1 for details.

### Personnel

None of the 67 hospitals had a specialist (a medical doctor who completed specialist training) in either general surgery, paediatric surgery or anaesthesiology. However, close to half (33%–49%) reported having general medical officers who performed surgery on children, including: 86% of Zambian hospitals (M = 1, range 0–5), 23% of Malawian hospitals (M = 0, range 1–3), and 42% of Tanzanian hospitals (M = 0, range 1–10)—see Table 2. Only one in five hospitals had nurses trained in paediatric care, and only two hospitals had a paediatrician. Notably, in the absence of anaesthesiologists, nurse anaesthetists were reported in 92% of hospitals in Tanzania (M = 3 range 0–4), in 24% of Zambian hospitals (M = 0, range 1–2) and in no district hospitals in Malawi (Table 2).

**Table 2:** Personnel available in district hospitals.

Number of district hospitals with:	Malawi (*n* = 22 DHs)	Zambia (*n* = 21 DHs)	Tanzania (*n* = 24 DHs)	TOTAL (*n* = 67)
	*N* (%)	*N* (%)	*N* (%)	*N* (%)
Paediatric surgeons	0 (0%)	0 (0%)	0 (0%)	0 (0%)
General surgeons	0 (0%)	0 (0%)	0 (0%)	0 (0%)
General medical officers (doing surgery on children)	5 (22.7%)	18 (85.7%)	10 (41.7%)	33 (49.3%)
Paediatricians	0 (0%)	1 (4.8%)	1 (4.2%)	2 (3.0%)
Paediatric trained nurses	6 (27.3%)	5 (23.8%)	3 (12.5%)	14 (20.9%)
Anaesthesiologists (MD)	0 (0%)	0 (0%)	0 (0%)	0 (0.0%)
Nurse anaesthetists	0 (0%)	5 (23.8%)	22 (91.7%)	27 (40.3%)

### Infrastructure, equipment and supplies

Tables 3–5 report a wide range of availability of infrastructure, fairly good availability of basic surgical equipment and supplies, and widespread shortages of specific paediatric care items. Certain basic infrastructure items were widely available, such as: hospital laboratory testing of blood and urine (88%), functioning x-ray (76%) and ultrasound machines (84%) and medical records department (100%). Over half of hospitals in Tanzania, few in Zambia and none in Malawi reported uninterrupted electricity supply, aggravated by lack of functional back-up electricity generators in one third of hospitals—see Table 3.

**Table 3:** Number of district hospitals with infrastructure always available.

	Malawi (*n* = 22 DHs)	Zambia (*n* = 21 DHs)	Tanzania (*n* = 24 DHs)	TOTAL (*n* = 67)
	*N* (%)	*N* (%)	*N* (%)	*N* (%)
Running water	10 (45.5%)	17 (81%)	22 (91.7%)	49 (73.1%)
External electricity	0 (0%)	4 (19%)	14 (58.3%)	18 (26.9%)
Functioning back-up generator	16 (72.7%)	10 (47.6%)	17 (70.8%)	43 (64.2%)
Incinerator	21 (95.5%)	18 (85.7%)	21 (87.5%)	60 (89.6%)
Medical records	22 (100%)	21 (100%)	24 (100%)	67 (100%)
Emergency department	3 (13.6%)	3 (14.3%)	4 (16.7%)	10 (14.9%)
Postoperative care area	3 (13.6%)	2 (9.5%)	12 (50%)	17 (25.4%)
Special care baby unit	10 (45.5%)	7 (33.3%)	5 (20.8%)	22 (32.8%)
Intensive Care Unit	0 (0%)	0 (0%)	0 (0%)	0 (0%)
Pre-tested blood available/blood bank	3 (13.6%)	3 (14.3%)	13 (54.2%)	19 (28.4%)
Lab to test blood & urine	16 (72.7%)	20 (95.2%)	23 (95.8%)	59 (88.1%)
Functioning x-ray machine	18 (81.8%)	18 (85.7%)	15 (62.5%)	51 (76.1%)
Functioning Ultrasound machine	17 (77.3%)	18 (85.7%)	21 (87.5%)	56 (83.6%)
Functioning CT scanner	0 (0%)	0 (0%)	0 (0%)	0 (0%)

**Table 4:** Number of district hospitals with equipment always available.

	Malawi (*n* = 22 DHs)	Zambia (*n* = 21 DHs)	Tanzania (*n* = 24 DHs)	TOTAL (*n* = 67)
	*N* (%)	*N* (%)	*N* (%)	*N* (%)
Oxygen compressed cylinder	16 (72.7%)	13 (61.9%)	18 (75%)	47 (70.1%)
Oxygen concentrator	19 (86.4%)	20 (95.2%)	20 (83.3%)	59 (88.1%)
Resuscitator bag valve & mask (paediatric)	20 (90.9%)	19 (90.5%)	22 (91.7%)	61 (91.0%)
Oropharyngeal airway (paediatric)	18 (81.8%)	16 (76.2%)	19 (79.2%)	53 (79.1%)
Endotracheal tubes (paediatric)	16 (72.7%)	14 (66.7%)	18 (75%)	48 (71.6%)
Anaesthesia machine	21 (95.5%)	16 (76.2%)	17 (70.8%)	54 (80.6%)
Pulse oximeter	21 (95.5%)	21 (100%)	21 (87.5%)	63 (94.0%)
Oxygen mask and tubing	22 (100%)	19 (90.5%)	24 (100%)	65 (97.0%)
Stethoscope	22 (100%)	21 (100%)	24 (100%)	67 (100.0%)
Blood pressure measuring equipment (paediatric cuffs)	5 (22.7%)	8 (38.1%)	14 (58.3%)	27 (40.3%)
Thermometer	21 (95.5%)	20 (95.2%)	24 (100%)	65 (97.0%)
Weighing scale for infants	22 (100%)	20 (95.2%)	22 (91.7%)	64 (95.5%)
Instrument sets (abdominal & c-section)	17 (77.3%)	17 (81%)	22 (91.7%)	56 (83.6%)
Kidney dish (stainless steel)	22 (100%)	20 (95.2%)	24 (100%)	66 (98.5%)
Sterilizer (autoclave)	15 (68.2%)	21 (100%)	21 (87.5%)	57 (85.1%)
Suction pump (manual or electric)	21 (95.5%)	20 (95.2%)	23 (95.8%)	64 (95.5%)
Electrocautery machine	6 (27.3%)	19 (90.5%)	11 (45.8%)	36 (53.7%)
Apnoea monitor	8 (36.4%)	3 (14.3%)	3 (12.5%)	14 (20.9%)
Syringe pump	7 (31.8%)	6 (28.6%)	4 (16.7%)	17 (25.4%)
Neonatal T-piece	4 (18.2%)	4 (19%)	9 (37.5%)	17 (25.4%)
Endoscopes (gastro, colon, broch)	3 (13.6%)	5 (23.8%)	5 (20.8%)	13 (19.4%)
Operating room lights	8 (36.4%)	11 (52.4%)	18 (75%)	37 (55.2%)

**Table 5:** Number of district hospitals with supplies always available.

	Malawi (*n* = 22 DHs)	Zambia (*n* = 21 DHs)	Tanzania (*n* = 24 DHs)	TOTAL (*n* = 67)
	*N* (%)	*N* (%)	*N* (%)	*N* (%)
Gloves (sterile)	19 (86.4%)	14 (66.7%)	22 (91.7%)	55 (82.1%)
Gloves (examination)	19 (86.4%)	15 (71.4%)	23 (95.8%)	57 (85.1%)
Nasogastric tubes 12F or smaller	8 (36.4%)	10 (47.6%)	17 (70.8%)	35 (52.2%)
Intravenous fluid infusion sets	18 (81.8%)	19 (90.5%)	23 (95.8%)	60 (89.6%)
Blood transfusion sets	21 (95.5%)	19 (90.5%)	24 (100%)	64 (95.5%)
IV cannulas	20 (90.9%)	19 (90.5%)	24 (100%)	63 (94.0%)
Syringes	20 (90.9%)	19 (90.5%)	24 (100%)	63 (94.0%)
Disposable needles	19 (86.4%)	21 (100%)	24 (100%)	64 (95.5%)
Tourniquet	3 (13.6%)	6 (28.6%)	22 (91.7%)	31 (46.3%)
Sterile gauze	12 (54.5%)	14 (66.7%)	24 (100%)	50 (74.6%)
Bandages sterile	7 (31.8%)	11 (52.4%)	21 (87.5%)	39 (58.2%)
Adhesive tape	16 (72.7%)	11 (52.4%)	24 (100%)	51 (67.1%)
Suture (absorbable)	11 (50%)	14 (66.7%)	24 (100%)	49 (73.1%)
Suture (non-absorbable)	11 (50%)	15 (71.4%)	24 (100%)	50 (74.6%)
Urinary catheters (size 6F)	0 (0%)	2 (9.5%)	7 (29.2%)	9 (13.4%)
Sharps disposal container	21 (95.5%)	20 (95.2%)	24 (100%)	65 (97.0%)
Scalpel blades	20 (90.9%)	19 (90.5%)	24 (100%)	63 (94.0%)
Facemasks	22 (100%)	14 (66.7%)	22 (91.7%)	58 (86.6%)
Eye protection (goggles, safety glasses)	11 (50%)	9 (42.9%)	19 (79.2%)	39 (58.2%)
Apron	19 (86.4%)	16 (76.2%)	21 (87.5%)	56 (83.6%)
Boots (theatre shoes)	18 (81.8%)	13 (61.9%)	23 (95.8%)	54 (80.6%)
Gowns (for surgeon/scrub nurse)	16 (72.7%)	16 (76.2%)	21 (87.5%)	53 (79.1%)
Drapes for operations	15 (68.2%)	15 (71.4%)	22 (91.7%)	52 (77.6%)
Chest tubes 12F or smaller	1 (4.5%)	5 (23.8%)	3 (12.5%)	9 (13.4%)
Trach tubes	3 (13.6%)	7 (33.3%)	8 (33.3%)	18 (26.9%)
Laparoscopic supplies	0 (0%)	0 (0%)	0 (0%)	0 (0%)

Most hospitals in Zambia and Tanzania, but less than half in Malawi had access to running water. A blood bank with pre-tested blood was available in over half (54%) of district hospitals in Tanzania and in only 14% of hospitals in Malawi and Zambia. Around 85% of hospitals lacked an emergency department (ED), two thirds lacked a special baby care unit, three quarters had no dedicated postoperative care area and none had an Intensive Care Unit (ICU).

Basic equipment such as stethoscopes, oxygen masks and tubing, pulse oximeters, thermometers, suction pumps and paediatric resuscitation equipment was always or almost always available across all three countries—see Table 4. While operating theatre equipment (instrument sets, electrocautery machines, lights and sterilisers) was generally available in Zambia and Tanzania, shortages were more marked in Malawi. Paediatric equipment such as endoscopes, neonatal T-pieces, syringe pumps and paediatric blood pressure cuffs were available in only 20%–30% of hospitals in Zambia and Malawi and were more common in Tanzania. Critically, most (72% of all) hospitals reported paediatric endotracheal tubes, essential for safe anaesthesia in children.

The infrastructure section of the survey tool provided data (not tabulated here) on the number of hospitals with newborn incubators, paediatric ventilators in ICU, and major operating rooms. Zambia had the lowest proportion of hospitals with newborn incubators (17%), followed by Malawi (50%) and Tanzania (67%), whose district hospitals, again, were best equipped. Only one of the 67 hospitals (in Zambia) reported having a paediatric ventilator.

Almost all hospitals reported the availability of surgical supplies for adults (see Table 5). Whereas, paediatric-specific surgical supplies were in short supply, with shortages twice as likely in Malawi compared with Tanzania: nasogastric tubes, sized 12F or smaller (36% in Malawi v 71% in Tanzania), chest tubes, 12F or smaller (5% v 13%), urinary catheters, size 6F (0% v 29%), and tracheostomy tubes (14% v 33%). Malawi had additional shortages of basic supplies such as tourniquets (14%), bandages (32%) and suture materials (50%), which were available in 88%–100% of hospitals in Tanzania.

## Discussion

Despite the attention drawn to global surgery by the 2015 Lancet Commission on Global Surgery (LCoGS) report, few initiatives have specifically focused on paediatric surgery (19). Also, the LCoGS report did not include any paediatric-specific indicators to monitor progress in global surgery, although half of the population in SSA are children (20). This has contributed to the apparent neglect of the burden of surgical conditions among children, especially in LMICs (21). Yet, in our 2017 study of cases that presented to eight district hospitals in Malawi and nine in Zambia, over a 24-month period, children 0 to 15 years accounted for two-thirds of trauma cases and 40% of hernias in girls that were managed in operating theatres (8). Hence, the scale of paediatric surgical care undertaken at district hospitals in some SSA countries is being ignored as much as neglected.

The first step in addressing this priority is to assess district hospital capacity to deliver paediatric surgical care safely. Our study provides evidence not previously available in SSA, based on a sample of 67 district-level hospitals in three African countries. It systematically collected data using a standardised tool employed by other studies (13), which can be replicated over time to monitor trends. Despite limitations of the tool—see below, it provides evidence on some of the strengths and weaknesses of country-level responses to this burden by triangulating the findings with other published evidence (18). Our study reported an overall higher PediPIPES index score than in a similar study in 39 district level facilities in Pakistan (22). The paediatric surgical capacity of district hospitals in our study, for the respective PIPES components, was similar to a study in West Africa, where 32 of 37 surveyed hospitals were tertiary, two were secondary, and district, private and mission hospitals were one each (13).

While the overall PediPIPES index scores were similar—5.8 in Zambia and Malawi and 6.2 in Tanzania, these scores masked some notable country-specific patterns and differences in the specific components. Infrastructure, equipment and supplies, including those specific to the surgical needs of children, were more widely available in district hospitals in Tanzania. Whereas, hospitals in Malawi, which scored lowest in these components, reported more widespread capacity to conduct a range of paediatric procedures. This could reflect the efforts to improve district-level surgical care in Malawi through innovative surgical training and supervision programmes designed specifically to address the population's surgical needs in rural areas (23).

The overall pattern of paediatric interventions and procedures reported for district hospitals across the three countries is similar and plausible, considering no paediatric surgeons were reported to practise at district level. Most district hospitals undertake minor emergency interventions and elective cases, especially covering orthopaedic procedures for trauma and abdominal surgery, consistent with our 2017 study (8). Surgical interventions are not reported for more complex congenital cases, which usually are referred for specialist care at higher level hospitals (24). However, the surgical referral systems in LMIC also face significant challenges (25). Future interventions tackling shortages of paediatric surgical capacity at district level must also address deficiencies along the patient referral journey.

Lack of trained staff has been considered one of the most important barriers to deliver safe surgery to children (26). In our study, there were medical doctors performing surgery on children. Although they were very limited in numbers in Malawi and Tanzania, they were present in 85.7% of Zambian hospitals. Only 5 of 22 sampled hospitals in Malawi reported a cadre (general medical officers) who undertook surgery on children and none reported anaesthesiologists or nurse anaesthetists that provided anaesthesia. Other studies done in the region showed that the bulk of surgical care at district level is delivered by medical doctors working alongside non-physician clinicians (27). Indeed, most of the district-level surgical and anaesthesia providers in Malawi, and many in Zambia are non-physician clinicians (NPCs) and some do manage paediatric surgical cases (8, 28). However, the PediPIPES tool in its current form is not designed to capture these cadres. Hence, the first lesson from this study is the need to modify facility assessment tools to ensure that paediatric surgical capacity surveys take account of the realities of who is delivering surgical and anaesthesia care to children at district hospitals in SSA.

Essential supplies such as paediatric size nasogastric tubes, urinary catheters and chest tubes were particularly lacking in Malawi, where more hospitals reported undertaking a wider variety of cases. Such shortages have a major negative effect on the readiness to perform surgery. A study in Malawi demonstrated that surgical supply shortages contributed to 51% of cancelled elective procedures (29). When essential infrastructure is unavailable, surgical outcomes are compromised (30). The evidence from our earlier study (18) suggests that district hospitals in Malawi, Zambia and Tanzania experience shortages of infrastructure, equipment and supplies, needed to safely deliver elective and emergency surgical care and administer anaesthesia to adults. The evidence from this study demonstrated that shortages in equipment and supplies specific to paediatric surgery are greater. This is worrying because children in need of surgical care undoubtedly present to these settings (8).

Since the inclusion of essential and emergency surgery as a key component of Universal Health Coverage (31, 32), and the work of the Disease Control Priorities 3 (DCP3) Country Translation project (32), several countries have included surgery in their essential packages of health services. In addition, countries with relatively high enrolment rates into health insurance schemes have included surgery in the health benefit packages of these schemes [e.g., Ghana, Kenya, Rwanda (33–36]). However, these packages do not specifically mention paediatric surgery, and paediatric surgery continues to be absent from most National Health Strategic Plans in SSA, despite the majority of the population being young (36).

Also, a list of cost-effective, essential surgical procedures for children is yet to be defined and included in essential health packages and national surgical and health plans. Of equal importance is the need to define the health systems level (national, referral and district hospital) at which each procedure should take place and the capacity conditions that need to be in place to do so safely (37). National surgical plans need to define the paediatric surgical responses and capacities that are required at all levels of the health system and, based on these, agree on lists of paediatric surgical procedures that are allowed and should be undertaken at national, referral and district hospital levels, the latter being the first level of surgical care in SSA (38).

The Global Initiative for Children's Surgery (39), an international interest group, has been advocating for inclusion of children's surgical care in national health and surgical plans. They have developed practical recommendations on how to do so, including general guidance on optimal resources for children surgery (40), to be adapted to each country's context. We support their recommendations, which are reinforced by the results in this paper, which have highlighted critical surgical capacity gaps at district level hospitals in MTZ, particularly around the paediatric surgical workforce. Countries such as Zambia and Tanzania, where the current NSOAP has (Zambia) or is about to expire (Tanzania), are well placed to integrate a district hospital response to children's essential and emergency surgical needs in the next NSOAP planning cycle.

## Limitations

Our study has several limitations. Firstly, there is potential selection bias and limited generalisability. In Tanzania, only hospitals in the Northern Zone (one of seven zones, countrywide) were surveyed, for feasibility reasons (17). Sampling in Malawi and Zambia was country-level representative. Secondly, as with previous studies that used the PediPIPES tool, facility staff-reported data were collected however, recall or ascertainment bias was reduced by having a minimum of two respondents for each facility. Thirdly, the facility respondents, at least two of whom worked in the facility's operating theatre, generally lacked specific paediatric surgical training, which could have impacted on the accuracy of their responses. The research team, however, had 5 + years prior experience of researching district hospital surgical facilities in two of the three studied countries, enabling the team to detect and resolve obvious inaccuracies during data cleaning, analysis and manuscript writing. Finally, the PediPIPES tool has deficiencies when assessing surgical capacity at district hospitals in SSA in particular, it does not capture non-specialist and non-physician cadres who undertake surgical interventions in children in these settings. One of the recommendations in this paper points to the need to adapt and utilise an improved tool for future use in SSA countries.

## Data Availability

The raw data supporting the conclusions of this article will be made available by the authors, without undue reservation.
